# 

**Published:** 1908-07

**Authors:** 


### CONTENTS.

ORIGINAL COMMUNICATIONS—
The Doctor and His Relation to Society. By J. C. Olmsted, M. D., Atlanta, Ga. .. .. .. 183
A Casual Review of the Work on the Appendix. By Willis Jones, M. D., Surgeon to Wesley Memorial Hospital, Atlanta, Ga. .. .. .. 189
Pyelitis in Pregnancy. By F. G. Hodgson, M. D., Gynaecologist to Wesley Memorial Hospital, Surgeon to Presbyterian Hospital, Gynaecologist and Abdominal Surgeon to Tabernacle Infirmary, Instructor in Gynaecology in Atlanta College of Physicians and Surgeons, Atlanta, Ga.. .. .. .. 204
Nervous Diseases Caused by Alcoholic and Metalic poisoning. By J. Cheston King, A. B., M. D., Atlanta, Ga .. .. .. 210
Higher Medical Education. By E. R. Anthony, M. D., Griffin, Ga. .. .. .. 216
Outdoor Life for the Prevention and Cure of Disease. By Paul Paquin, M. D., Member American Medical Association, North Carolina State Local, etc., Superintendent Asheville-Biltmore Sanitarium, Asheville, N. C... .. .. .. 219
CORRESPONDENCE—
Letter from the Juvenile Protective Association .. .. .. 224
EDITORIALS—
The Chicago Meeting of the American Medical Association .. .. .. 226
Devise Plans for Regulating Practice of Medicine. .. .. .. 227
Mortality Statistics .. .. .. 228
The Need of Better Water and Better Sewers .. .. .. 229
Fritz Schaudinn .. .. .. 250
Medical Aid and Nursing for the Poor .. .. .. 231
BOOK REVIEWS.. .. .. .. 231
SELECTIONS AND ABSTRACTS.. .. .. .. 234
NEWS AND NOTES .. .. .. .. 237

